# Long term outcomes after bare metal stent implantation

**DOI:** 10.1016/j.ihj.2021.10.004

**Published:** 2021-10-21

**Authors:** S. Harikrishnan, Rajesh Muralidharan, Avinash Mani, G. Sanjay, Srinivasa Prasad, S. Bijulal, S. Sivasankaran, Ajit Kumar Valaparambil

**Affiliations:** Department of Cardiology, Sreechitra Tirunal Institute for Medical Sciences and Technology, Thiruvananthapuram, Kerala, 695011, India

**Keywords:** Coronary artery disease, Bare metal stents, Target vessel revascularisation

## Abstract

Long term outcome data after BMS implant is not available from the Indian subcontinent. This is a prospective observational study which aims to study long term outcomes after BMS implant at a tertiary care centre. 100 consecutive patients underwent BMS implant and were followed up for 20 years. LAD was the most common vessel involved and different types of BMS were implanted. All-cause mortality was noted in 21% (n = 21) whereas cardiac mortality was seen in 16% (n = 16). Cumulative revascularisation free survival at 20 years was 71%. The study showed that long term outcomes after BMS implant were fare and acceptable.

## 1 Introduction

Stent implantation has been associated with superior results as compared to balloon angioplasty in coronary stenosis.^1^ The initial stent design comprised of bare metal stents (BMS) which provided a scaffold and reduced restenosis rates. The use of BMS has reduced with the advent of drug eluting stents (DES). Currently, BMS is primarily used in patients with high bleeding risk who cannot tolerate long term dual antiplatelet therapy (DAPT). Continued long term benefits after BMS implantation has been documented from the West.^2^ However, there is paucity of long-term outcome data from the Indian subcontinent. In this study, we aim to evaluate long term outcomes (>10 years) after BMS implantation at a tertiary care centre in India.

## 2 Methods

This is a prospective observational single centre performed during the period 1996 to 1998. 1st 100 consecutive patients who had a BMS implanted at our centre were included in the study. Patients undergoing primary PCI and those requiring adjunctive therapy (Eg. atherectomy) were excluded from the study. All patients underwent revascularisation of culprit vessel. In case of multivessel disease, non-culprit vessel revascularisation was based on operator discretion. Coronary stenting was done using standard techniques and all patients received DAPT after procedure for a minimum period of 4 weeks.

The patients were regularly followed up on out-patient basis or telephonic consultations. Data regarding mortality, myocardial infarction, angina, repeat angiogram, target vessel revascularisation (TVR), target lesion revascularisation (TLR), non-target vessel revascularisation (NVR) and in-stent restenosis (ISR) on follow-up was obtained.

Analysis of data was done using SPSS v23 (IBM, Armonk, NY). Continuous variables were expressed as mean ± SD and categorical variables as proportions. Kaplan–Meier analysis was done to determine event free survival rates.

## 3 Results

100 patients with BMS implant were included in the study. The baseline characteristics of the patients are described in Table 1. Majority of patients were males with high prevalence of cardiovascular risk factors. ST-elevation myocardial infarction was the most common clinical presentation (52%). Majority of patients had single vessel disease with left anterior descending artery (LAD) involvement being most common (67%). Different types of BMS were used (NIR 27%, WIKTOR 16%, PALMAZ SCHATZ 15%, BARD 8%). The average number of stents used per patient was 1.06 ± 0.01 with a mean stent length of 17.68 ± 5.86 mm. Mean stent diameter used was 3.07 ± 0.38 mm.

**Table 1:** Baseline characteristics of patients included in the study (n = 100). LVEF- Left Ventricular Ejection Fraction; STE-ACS- ST elevation Acute Coronary Syndrome, Non STE-ACS- Non ST Elevation Acute Coronary Syndrome; PCI- Percutaneous Coronary Intervention; POBA- Plain old balloon angioplasty.

Age (mean) (years)	51 ± 8.7
Male (%)	96%
Risk factors	
Diabetes	58%
Hypertension	49%
Dyslipidaemia	57%
Smoking	41%
Diagnosis	
STE- ACS	52%
Non STE-ACS	10%
Stable Angina	38%
LVEF (mean) SD	54 ± 4%
Extent of disease	
Single vessel disease	61%
Two vessel disease	29%
Triple vessel disease	10%
Multi-vessel PCI	1%
Graft Interventions	0
Previous POBA1%	15%
Previous CABG1%	1%

**Fig. 1:**
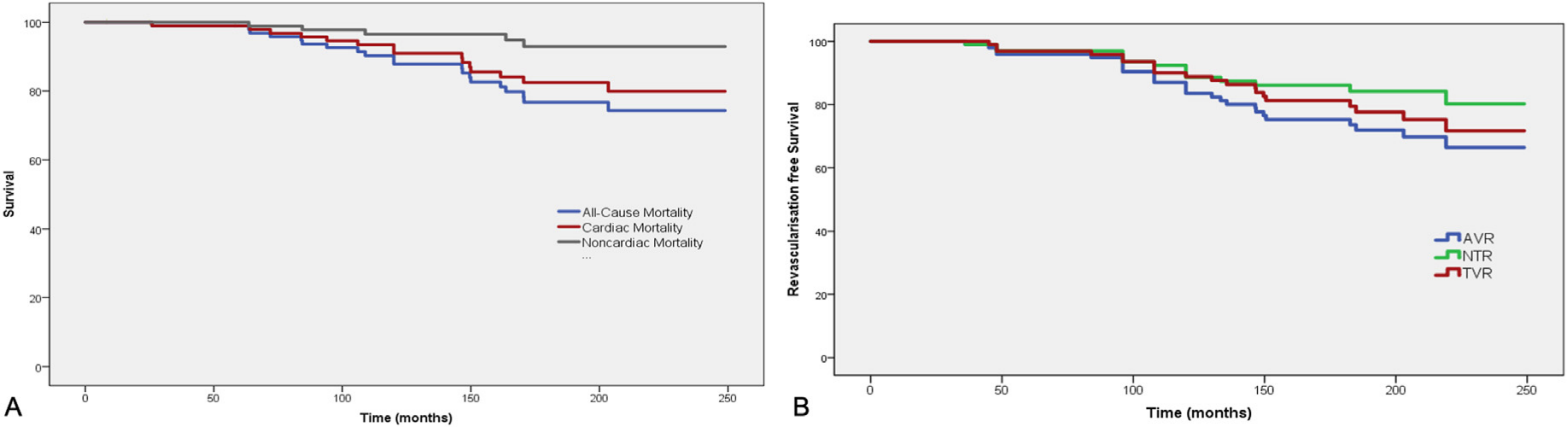
A. Kaplan Meir curve showing overall survival along with cardiac and non-cardiac mortality. B. Kaplan Meir curves showing revascularisation free survival on follow up.

99% patients (n = 99) were on regular follow up till 20 years after index procedure. All-cause mortality was noted in 21% (n = 21) of the study population whereas cardiac death was noted in 16% (n = 16). In patients who had cardiac mortality, 9 patients had acute MI whereas 7 patients suffered sudden cardiac death (SCD). 11% patients (n = 11) had documented MI on follow up. Recurrence of angina was noted in 62% (n = 62) patients on follow up amongst whom 41% patients had a positive stress test. Subsequently, 55% patients (n = 55) underwent CAG. ISR was noted on CAG in 23% patients (n = 23). 13% patients (n = 13) had TLR whereas TVR was seen in 23% (n = 23). NVR was noted in 16% of the study cohort (n = 16). The cumulative survival free of cardiac mortality at 20 years was 84% whereas cumulative revascularisation free survival at 20 years was 71% (Fig. 1).

## 4 Discussion

This was a prospective observational study where we aimed to study the long-term outcomes after BMS implant in a tertiary care centre in India. Majority of patients in study cohort were middle aged males with conventional risk factors. STEMI was the commonest presentation and LAD was the most common vessel involved. On long term follow up (20 years), mortality free survival was noted to be around 79% whereas revascularisation free survival was seen in 71% of the study cohort.

There are very limited studies from India which address the long-term outcomes after BMS implant, in contrast to Western literature. Fuji et al demonstrated 10-year survival and event free survival in 73.6% and 39.2%, respectively, in a Japanese cohort of 125 patients.^3^ Another study by Yamaji et al demonstrated 15-year survival rates of 56.4%.^4^ The 20-year survival rates in our study (79%) were similar to the outcomes shown by Fuji et al whereas revascularisation rates in our study was significantly lower. This could be primarily attributed to the heterogenous population group included in the study.

Restenosis and repeat revascularisation are the Achilles heel of PCI therapy. In the BENESTENT trial, 5 year outcome data revealed repeat revascularisation rate of 17.2%.^2^ Yamaji et al reported 15-year revascularisation rate of 71.8% amongst whom 36% had target lesion revascularisation (4). The current study showed significantly lower rates of TLR and TVR which could be attributed to the baseline lesion characteristics. BMS are prone to develop in-stent restenosis in long term due to neointimal hyperplasia.^5^ Increase in TLR and TVR was noted in the current study after 1 year of implant which stabilized on long term follow up (>10 years). This was in accordance to the observations by Choussat et al which demonstrated stability of TLR at 8–10 years after BMS implant.^6^ The increase in TLR rates noted corresponds to the late re-narrowing phase demonstrated by Kimura et al in BMS implants after 4 years follow up.^7^

NVR rates, beyond 1 year, were noted to be higher (15%) as compared to TLR (8%) in the present study. This observation was similar to the findings noted in studies with intermediate term outcomes (3–10 years) after BMS implant.^8^^,^^9^ This demonstrates that luminal narrowing progresses in non-target vessels progresses in a similar manner and highlights the need for strict risk factor control. The current study shows acceptable long-term survival and event free rates in patients with BMS and results are comparable with the available Western data.

The study has its inherent limitations, with it being a single centre study. Data regarding risk factor profile and medications on follow up were not available for all patients. Inherent selection bias could also influence the results.

## 5 Conclusion

Patients with BMS implant fare well in the long term with acceptable survival and repeat revascularisation rates. Late revascularisation was more commonly noted in non-target vessels whereas stented segments remained stable over time.

## Funding

Nil.

## Declaration of competing interest

The authors do not have any conflict of interests to declare.
